# Growth-rate dependent response of mycobacteria to relief of inhibition by a bacteriostatic antibiotic

**DOI:** 10.1093/femsle/fnag048

**Published:** 2026-04-20

**Authors:** Priyanka Chauhan, Frank J Bruggeman

**Affiliations:** Systems Biology Lab, A-Life, AIMMS, VU University, De Boelelaan 1087, 1081 HV Amsterdam, The Netherlands; Systems Biology Lab, A-Life, AIMMS, VU University, De Boelelaan 1087, 1081 HV Amsterdam, The Netherlands

**Keywords:** serine hydroxamate, mycobacteria, antibiotic persistence, lag-time, growth rate, regrowth

## Abstract

Pathogenic bacteria generally resume growth upon removal of bacteriostatic antibiotics. However, it is poorly understood how pre-growth conditions influence (i) the lag time before growth resumption and (ii) induction of antibiotics tolerance upon removal of the antibiotics. To study these traits in *Mycobacterium smegmatis*, we used a stringent-response-inducing bacteriostatic inhibitor, serine hydroxamate (SHX), which mimics a growth-rate-reducing antibiotic. We find that SHX-induced, growth-arrested cells display characteristics typical of persister cells, i.e. low ATP levels, increased antibiotic tolerance, and delayed growth-recovery. Single-cell microscopy shows morphological changes in SHX-treated cells (elongation and polar swelling) and growth resumption upon SHX removal after a lag time. During this lag time, the cells displayed increased antibiotic survival, which remained evident after complete recovery from SHX stress. Varying the SHX dose, exposure time, and pre-exposure growth rate indicated that the duration of the lag phase depends on the SHX exposure time and the prior growth rate, not on the SHX dose. The lag phase could be shortened by over two-fold with the addition of amino acids, pyruvate, or spent medium, indicating that growth inhibition induces a relievable metabolic bottleneck. Our findings align with studies of other (evolutionarily distant) microorganisms, suggesting that mycobacteria may be limited by similar phenotypic trade-offs associated with adaptive responses to sudden growth rate reductions.

## Teaser

The pre-growth condition of *Mycobacterium smegmatis* influences its recovery from slow growth rate, and medium supplements can speed up recovery.

## Introduction

The genus mycobacteria, containing several pathogenic and opportunistic pathogenic species, present a formidable challenge to global health, with tuberculosis (TB) ranking among the most prevalent and deadly infectious diseases (WHO 2021). Despite advancements in anti-mycobacterial drug development, managing mycobacterial infections remains a daunting challenge. A key contributing factor to this challenge is the existence of persister cells within mycobacterial populations (Wayne and Sohaskey 2001, Zhang 2014, Sarathy et al. 2018). These cells, characterized by slow or non-growing states, exhibit remarkable tolerance to conventional antimicrobial agents without genetic resistance (Keren et al. 2004, Lewis 2010, Helaine et al. 2014). These dormant bacteria can lead to asymptomatic persistent infections that often go undiagnosed, contributing to treatment failures, prolonged courses, increased drug resistance, and relapses (Keren et al. 2004, Lewis 2007, Lewis et al. 2016, Lewis and Shan 2017, Levin-Reisman et al. 2017). Thus, understanding the mechanisms behind persister cell formation is crucial for developing innovative therapeutic strategies to target and eliminate these resilient subpopulations, thereby improving treatment outcomes for mycobacterial-related diseases and other bacterial persistent infections.

Various environmental stresses, such as sub-lethal antibiotic concentrations, heat shock, oxidative stress, nutrient deprivation, iron limitation, low pH, biofilm formation, and host immune signals, can induce or modulate persister cell formation (Balaban et al. 2004, Amato et al. 2013 ). Since bacterial populations face these stresses, which are qualitatively different and often occur simultaneously, how each of them can induce persister cell formation remains puzzling (Lewis 2010). A common feature of stress responses is that they are all associated with a sudden growth rate decrease, suggesting that this common response may underlie the induction of persister cell formation upon a cue (Pontes and Groisman 2019, Berkvens et al. 2022). The correlation between growth rate and antibiotic tolerance has been confirmed in several studies (Tuomanen et al. 1986, Brown et al. 1990, Łapińska et al. 2022).

Mycobacteria are constantly facing environmental changes. For instance, during the initial stages of infection, *M. tuberculosis* encounters nutrient-rich environments in the lung alveoli, whereas as the infection progresses and granuloma forms, they face a nutrient-limited environment (Berney and Cook 2010, Chandra et al. 2022). Since bacterial fitness depends not only on their ability to grow rapidly but also on their ability to survive when conditions worsen, it is vital to understand how bacteria adapt to a new environment and how this is influenced by past environmental cues. In *Escherichia coli*, it has been recently demonstrated that the rate of bacterial adaptation is not solely dependent on the inherent genetic capacity for mutations, but, notably, on the growth rate as well (Biselli et al. 2020). *Escherichia coli* cells have shown to survive longer in carbon starvation if they previously grew slower (Biselli et al. 2020). In another study, adaptation of *E. coli* to a gluconeogenic carbon source correlates negatively with their growth rate on a previous glycolytic carbon source (Kotte et al. 2014). Corroborating this study, Basan et al. reported that previously fast-growing *E. coli* cells took a relatively long time to adjust to the new medium, whereas slow-growing cells resumed growth much more quickly (Basan et al. 2020). Additionally, the duration of stress exposure has been found to play a pivotal role in shaping the bacterial adaptive response, which ultimately also effects persister formation; ageing of cell cultures has been shown to delay regrowth after reinoculation into fresh media (Luidalepp et al. 2011); the age of inoculum has shown to influence persister frequency, with late-stationary phase culture of *E. coli* with more persister cells than early-stationary phase culture (Luidalepp et al. 2011). Similar results have been found with antimicrobial treatment, where prolonged treatment periods led to wide lag-time distributions on recovery (Levin-Reisman et al. 2010, Fridman et al. 2014, Kaplan et al. 2021). Furthermore, the manifestation of large lag phases upon exposure to a new environment has been attributed to metabolic bottlenecks, where the rewiring of cellular processes and adjustment to new metabolic demands impede immediate growth (Basan et al. 2020). Therefore, understanding the interplay between growth rate, stress duration, and metabolic bottlenecks in bacterial adaptation is fundamental for elucidating the dynamics of bacterial response to environmental changes and for developing effective strategies to combat bacterial resistance and persistence.

Given that most studies on bacterial adaptations have been conducted using model organisms like *E. coli*, it is crucial to explore whether mycobacteria respond similarly or differently to these challenges. Therefore, we explore how mycobacteria respond to a sudden reduction in growth and what impact it has on their recovery. In addition, we investigated how the prior growth rate influences the recovery from sudden environmental stress and if the recovery could be fastened using different external stimuli.

## Materials and methods

### Bacterial strains, media, culturing conditions, and chemicals

For standard culturing, *Mycobacterium smegmatis* mc^2^ 155 (*M. smegmatis*) was grown at 37°C under shaking conditions in Middlebrook 7H9 media containing 0.05% Tween-80 supplemented with 0.5% albumin, 0.2% dextrose, and 0.085% NaCl, referred to as 7H9-ADS media. A standard culturing procedure was always used for each experiment: a pre-culture from stocks of *M. smegmatis* stored at −80°C was first fully grown. The pre-culture was then diluted to 1:1000 in fresh media and grown till exponential phase (∼0.4 OD_600_). This culture was then grown in the required growth media and conditions and then used for the experiment. To starve the cells, the bacterial cells of 0.2 OD_600_ were kept in PBS (Phosphate-Buffered Saline) containing 0.02% tyloxapol, a non-hydrolyzable detergent. To evaluate the effect of growth rate on recovery time after serine hydroxamate (SHX, CAS number: 55779-32-3, Sigma–Aldrich) treatment, cells were grown up to exponential (∼0.2 OD_600_) or stationary (∼3 OD_600_) phase in 7H9-ADS media. To grow under various carbon sources, 7H9 media containing 0.05% Tween-80 added with either glucose (0.2%) or glycerol (0.4%) or pyruvate (0.4%) was used. When we needed to plate *M. smegmatis* cells on agar, 7H9 agar plates supplemented with 10% (vol/vol) ADC enrichment (Albumin–Dextrose–Catalase, Difco) and 0.4% activated charcoal were used.

### SHX treatment of *M. smegmatis* cells

To evaluate the effect of SHX on the growth of *M. smegmatis*, the bacterial culture was grown till 0.2 OD_600_ in a 96-well plate in a Middlebrook 7H9-ADS medium, and then different concentrations of SHX (0–1500 µg/ml) were added. Growth was then monitored by measuring OD_600_ over a period of 72 h in a spectrophotometer (SpectraMax Plus 384, Molecular Devices, San Jose, CA, USA). Bacteria grown under various conditions of growth: up to exponential- or stationary-phase and under different carbon sources such as glycerol, glucose, and pyruvate, were diluted to an OD_600_ of 0.2 in fresh 7H9-0.05% Tween-80 media containing SHX (1000 µg/ml) and respective carbon source, and incubated till desired time duration in 96-well plate at 37°C under shaking conditions in a spectrophotometer.

### Total cellular ATP measurement assay

ATP levels of untreated and SHX-treated cultures of *M. smegmatis* were measured using the luciferin/luciferase system (ATP Bioluminescence Assay Kit HS II; Roche Applied Science), according to the manufacture's protocol. Briefly, after 60 h of SHX treatment, 100 µl sample was taken out, centrifuged, and resuspended in 100 µl dilution buffer. The same volume of lysis buffer was added to the sample to disrupt the cells. After removal of cell debris by centrifugation, the supernatant was transferred to a new tube. Then, 50 µl of sample was taken and transferred to a microtiter plate with the same volume of luciferase reagent added, and luminescence was measured using Synergy H1 spectrophotometer. The ATP concertation was estimated using the standard curve. The exponentially growing culture without SHX was used as untreated control and the same steps were performed with it. The CFU counts were also estimated for the SHX-treated and untreated samples, and ATP concentration was normalized with CFU count. Finally, the ATP amount is expressed as moles/CFU (presuming that the lysis efficacy was 100%).

### Antibiotic tolerance assay

To assess bacterial growth and survival after antibiotic treatment for SHX treated cultures, the culture was first centrifuged at 12 000 rpm for 5 min, washed and resuspended in fresh growth media. The cultures were then diluted to OD_600_ 0.01, and were treated with 1.25 µg/ml of ciprofloxacin (MIC = 0.5 µg/ml) and 1.25 µg/ml streptomycin (MIC = 0.25 µg/ml) followed by incubation at 37°C under shaking conditions. At the indicated antibiotic exposure time, the samples were collected and serially diluted (10-fold, 10^0^–10^−6^) and spotted on 7H9-ADC agar plates supplemented with 0.4% activated charcoal. The plates were incubated for 3–4 days at 37°C to determine surviving CFU counts.

### Growth recovery analysis

To recover the cells from stress, either SHX treatment or starvation, the cells were harvested (in 200 µl), and centrifuged at 12 000 rpm for 5 min, washed and resuspended in fresh growth media (in 200 µl) for regrowth (resulting in at least a 200-fold dilution of residual SHX upon resuspension). The regrowth was monitored by measuring OD_600_ in a spectrophotometer. For different carbon sources, the respective carbon source was added in recovery growth media. To calculate the recovery time, the time required for the initial inoculum density to double was calculated. Since with glycerol the longest lag phase was observed, this culturing condition was used to evaluate for the reduction of lag phase with addition of supplements to regrowth media. MP amino acid mix (4 g/l), or sodium pyruvate (10 mM) was supplemented to regrowth media to reduce lag phase. To evaluate the effect of spent media, the cells were resuspended in spent media after washing off SHX. To prepare spent media, supernatant was obtained from *M. smegmatis* cultures grown in 7H9 media containing 0.05% Tween-80 added with glycerol (0.4%) up to 0.4 OD_600_. The supernatant was sterilized by passage through a 0.22 μm filter before use.

### Single-cell microscopy observation

For time-lapse microscopy analysis, the procedure was followed as described earlier (Chauhan et al. 2022), with minor changes. Briefly, the agarose pads were prepared using low-melting-point agarose [1.5% (w/v)] dissolved in 7H9-0.05% Tween-80 media. Once cooled down, 10% of ADC enrichment was added. Gently, 700 µl of agarose was dispensed onto the glass cover slip (18 × 18 mm) placed in a sterile petri dish on the flat surface. The second cover clip was placed on the top to create an agarose sandwich. The petri dish was closed and left for ∼30 min to let the agarose solidify. The upper coverslip was then removed gently using a sterile scalpel blade and the required size of agarose pad was cut out. Then, the 2 µl of SHX treated and untreated bacterial sample (∼4000 cells/µl) was pipetted on top of the pad. After allowing cells to get adsorbed for 5–10 min, the agarose pad was flipped onto the glass slide. The lid of the slide was closed and sealed with parafilm to minimize drying of agarose pads. A drop of immersion oil was placed on the 100 X objective and the slide was mounted on the microscope stage. The imaging was carried out in time-lapse manner using a Nikon Ti-eclipse microscope (Nikon, Minato, Tokyo, Japan) at 37°C equipped with a TuCamsystem (Andor, Belfast, Northern Ireland) and 2 Andor Zyla 5.5 sCMOS Cameras (Andor) and a SOLA6-LCR-SB light source (Lumencor, Beaverton, OR).

### Statistical analysis

All experimental data are represented as mean ± SD, and statistical significance was determined using the unpaired two-tailed Student’s *t*-test without assuming a consistent SD followed by Holm–Sidak post-tests with *α* = 0.05 in GraphPad Prism v8.0 software. *P* values < 0.05 were considered statistically significant and represented as *P < 0.05, **P < 0.01, ***P < 0.001, and ****P < 0.0001.

## Results

### Serine hydroxamate induces bacteriostatic effect and increases tolerance in *M. smegmatis*

**Figure 1:**
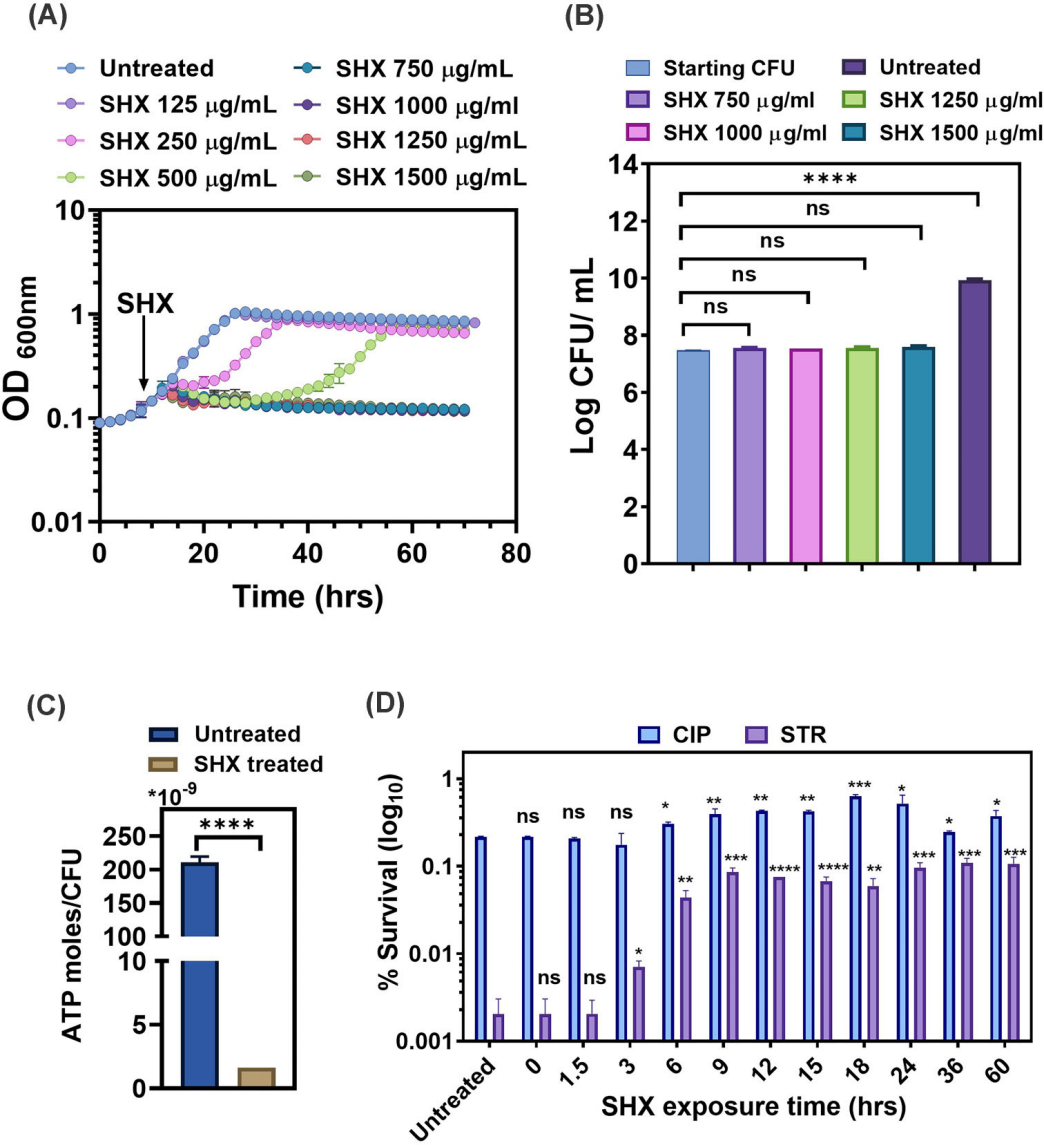
Effect of SHX on growth and antibiotic tolerance of *M. smegmatis*. (A) Optical density (OD_600_ nm) measurement of growth of *M. smegmatis* in the presence and absence of different concentrations of SHX. The Mean ± SD of three biological replicates are plotted. The arrow indicates the OD (∼0.2) at which the SHX was added. (B) Colony-forming unit (CFU) analysis for quantification of surviving *M. smegmatis* after exposure to indicated SHX concentrations for 60 h. Data are Mean ± SD from two independent experiments, each comprising three technical replicates. (C) ATP level measurement of *M. smegmatis* after treatment with SHX for 60 h. ATP levels were determined using the bioluminescence method. The exponentially growing culture without SHX was used as an untreated control. The Mean ± SD of two biological replicates, each comprising three technical replicates, are shown. (D) Surviving fraction (%) of *M. smegmatis* after 12 h treatment with ciprofloxacin (1.25 µg/ml) or streptomycin (1.25 µg/ml). SHX-treated cultures were washed and then diluted to OD₆₀₀ 0.01 in fresh medium and treated with antibiotics. Exponentially growing cultures without SHX were used as an untreated control. The Mean ± SD of three biological replicates, each comprising three technical replicates, are plotted. *P < 0.05, **P < 0.01, ***P < 0.001, and ****P < 0.0001; ns, not significant.

The activation of the stringent response, which is in many bacteria mediated by accumulation of the alarmone (p)ppGpp (Cashel and Gallant 1969, Prusa et al. 2018, Irving et al. 2021), generally induces slow growth (Amato et al. 2013, Pontes and Groisman 2019). The serine analogue, SHX, has often been used to induce stringent response in various bacteria (Tosa and Lewis 1971, Amato et al. 2013, Pontes and Groisman 2019). It mimics amino-acid deprivation by preventing seryl-tRNA from being charged and promotes the synthesis of (p)ppGpp by the protein RelA (Tosa and Lewis 1971, Goldman and Jakubowski 1990, Wendrich et al. 2002). However, with mycobacteria, contrasting results have been obtained by different studies, regarding the effectiveness of SHX in inducing stringent response (Primm et al. 2000, van de Weerd et al. 2016). A study by Weerd et al. suggested a role of the composition of the growth medium, showing that catalase could inactivate the effect of SHX (van de Weerd et al. 2016). Therefore, to assess the effect of SHX on the growth of *M. smegmatis*, we utilized catalase-free 7H9-ADS (albumin, dextrose, and sodium chloride complex) growth medium. We exposed exponentially growing *M. smegmatis* cultures to different concentrations of SHX (125-1500 µg/ml). We found that SHX inhibits growth in a concentration-dependent manner (Fig. 1A). Whereas intermediate concentrations of SHX (250 and 500 µg/ml) gave rise to temporary growth arrests, higher concentrations (750–1500 µg/ml) led to complete growth inhibition. Since no decrease in CFU count was observed after treatment with SHX (Fig. 1B and Fig. S1A), this indicates that SHX has a bacteriostatic effect and that growth arrest is not due to a lethal effect of SHX.

Next, we sought to determine whether the SHX-mediated growth arrest is related to increased mycobacterial tolerance towards antibiotics. Since persister cells are characterized by a lower energy state (Conlon et al. 2016, Shan et al. 2017, Wang et al. 2018), we tested the ATP levels after SHX treatment. Indeed, SHX-treated cells showed ∼300 times lower ATP levels compared to untreated cells (Fig. 1C). However, because ATP was measured at the population level, we cannot exclude the possibility that the antibiotic-surviving subpopulation maintains higher ATP levels than the culture average. Furthermore, we tested SHX-treated cells for antibiotic tolerance. After treatment with SHX, the cells were exposed to ciprofloxacin, which inhibits DNA replication by inhibiting DNA gyrase, and streptomycin, which inhibits protein synthesis by binding to the 30S subunit of the ribosome. Our results showed that cultures treated with SHX displayed increased survival against streptomycin (∼10– 100-fold increase when compared with an untreated culture; Fig. 1D). The survival first increased sharply with the exposure time of SHX and saturated after 9 h (Fig. 1D). We observed only a modest difference in survival level with ciprofloxacin between the SHX treated and untreated cultures (Fig. 1D). It is known that the stringent response provides partial protection from ciprofloxacin (Pontes and Groisman 2019), because it inhibits only new chromosomal replication events (Levine et al. 1991), whereas the pre-existing replication forks can be still sensitive to the gyrase-inhibiting effect of ciprofloxacin (Pontes and Groisman 2019). Together, the results presented above demonstrate that the growth arrest caused by SHX is associated with increased antibiotic tolerance.

### SHX-induced growth-arrested cells exhibit a lag in regrowth and maintain antibiotic tolerance after removal of SHX

**Figure 2:**
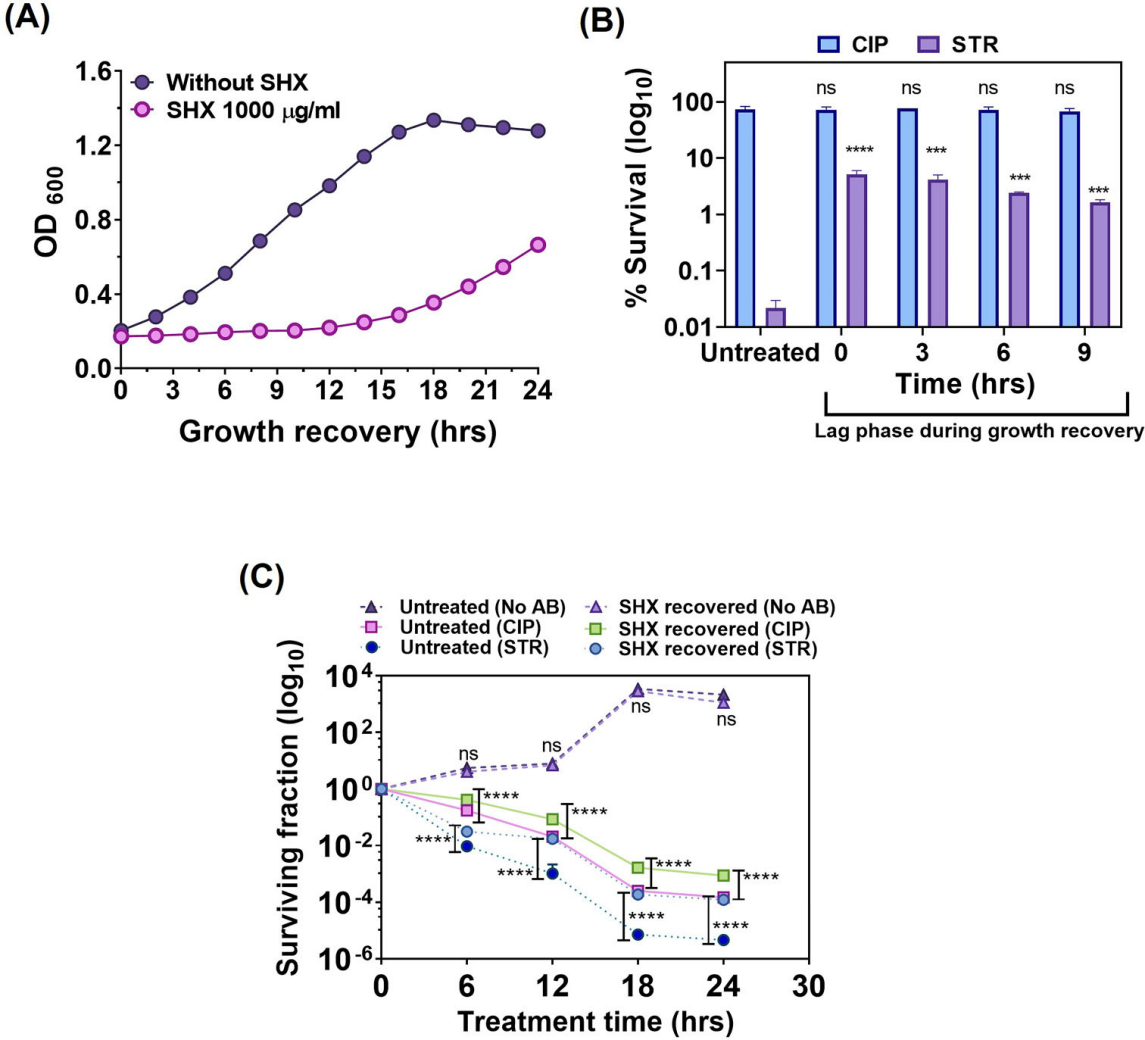
Recovery and antibiotics tolerance of *M. smegmatis* cells after removal of SHX. (A) Analysis of growth recovery of *M. smegmatis* in 7H9-ADS media following a 12-h treatment with SHX. The washing procedure was used, ensuring that the recovery lag is not attributable to residual SHX. The Mean ± SD of three biological replicates are plotted. (B) Measurement of surviving fraction (%) of *M. smegmatis* after treatment with 1.25 µg/ml of Ciprofloxacin (CIP) and 1.25 µg/ml Streptomycin (STR) at indicated time points of lag phase during growth recovery after removal of SHX. The duration of optical density constancy is marked as lag phase within the recovery phase. Notably, the treatment time for antibiotics was kept shorter (4 h) than the total lag phase time (∼10 h). The exponentially growing culture without SHX was used as an untreated control. The Mean ± SD of three biological, each with three technical replicates, are plotted. (C) Antibiotic tolerance assay of recovered *M. smegmatis* cultures (till exponential growth) after removal of SHX. The cells are treated with 1.25 µg/ml of CIP and 1.25 µg/ml of STR for indicated time and surviving fraction was measured. The Mean ± SD of three biological, each with three technical replicates, are plotted.

We next examined the impact of the sudden growth arrest (induced by SHX addition) on the recovery of growth by *M. smegmatis* cells following SHX removal. After treatment of an exponentially growing culture of *M. smegmatis* with SHX for 12 h, the cells were washed, transferred to fresh growth media, and their growth recovery was monitored. The culture displayed an extended lag time (∼10 h) before resumption of growth (Fig. 2A). Observing the recovery of growth at individual cell level with single-cell time-lapse microscopy revealed cell-morphological changes upon SHX treatment (Fig. S2). The cells appeared elongated with swelling at their poles. In total, 37 single cells were tracked after SHX removal. Of which, 34 (91.9%) exhibited visible polar swelling, and 32 of them were able to regrow after a lag period within the experimental duration, indicating their viability (Fig. S2). The three cells without swelling showed no detectable activity, suggesting pre-existing non-viability; importantly, nearly all swelling cells were capable of regrowth.

Next, we determined whether the cells exhibit increased antibiotic survival during the extended lag phase following the removal of SHX. To assess this, post-SHX removal, we exposed cells to ciprofloxacin and streptomycin at different time intervals within the lag phase. Similar to increased antibiotic survival, observed with the SHX-induced growth-arrested culture (Fig. 1D), the cells displayed significantly increased survival fraction against streptomycin, whereas no difference in survival against ciprofloxacin was observed between untreated and SHX-treated cultures (Fig. 2B and Fig. S1B).

Next, to determine whether this increased tolerance against antibiotic is retained after growth resumes, the SHX-treated culture was regrown to exponential growth following SHX removal, and subsequently exposed to ciprofloxacin and streptomycin for varying duration. The surviving fraction was quantified by using CFU counting (Fig. 2D). The regrown cells showed higher tolerance and significantly increased persister fraction as compared to untreated cultures (Fig. 2C).

Together, these findings indicate that bacteria maintain an enhanced tolerant state even after stress removal.

### SHX exposure duration determines the recovery time, not treatment concentration

**Figure 3:**
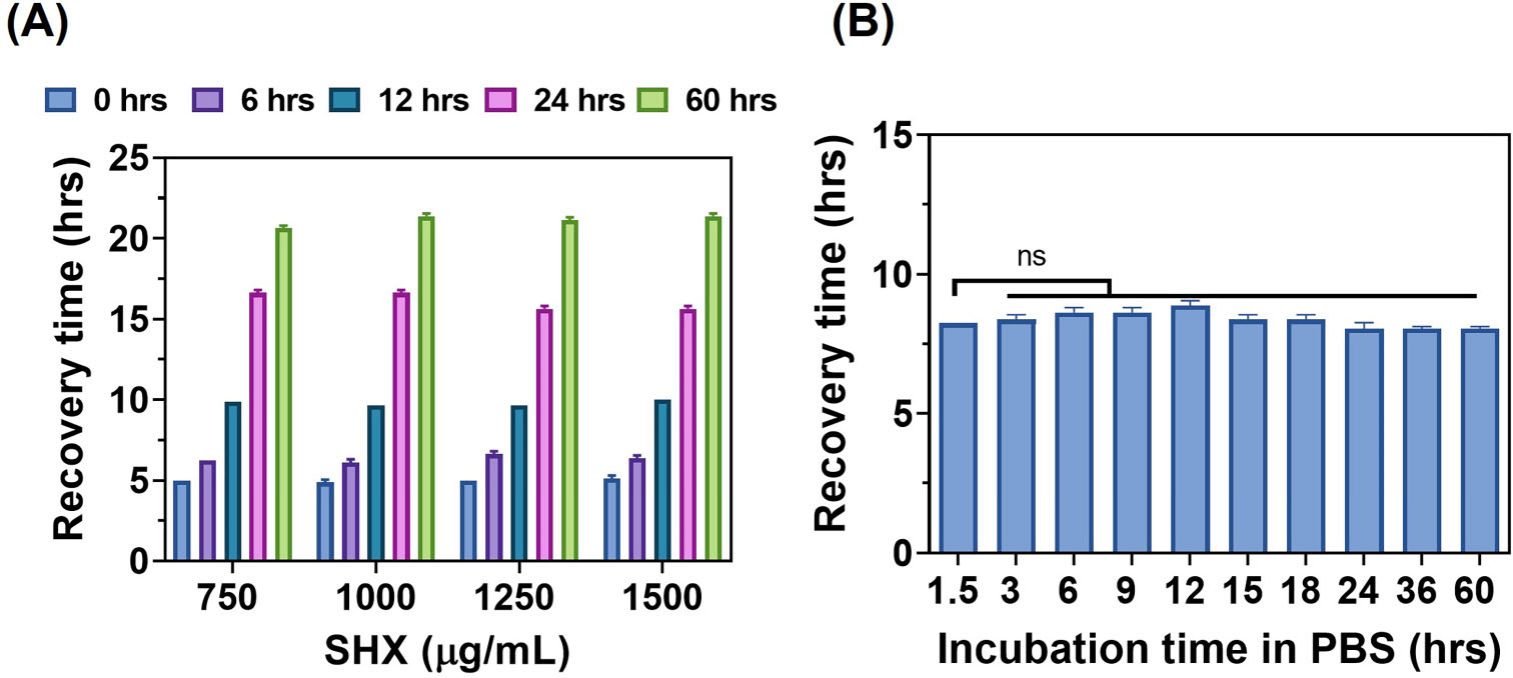
Effect of different exposure duration and concentration of SHX, and starvation in PBS on recovery time of *M. smegmatis*. (A) Dependence of SHX exposure duration and concentration on the recovery time. After treatment with indicated concentrations and exposure time of SHX, *M. smegmatis* cells were grown in 7H9-ADS media for growth recovery. We define the recovery time as the time required for a population to double its initial optical density in response to indicated SHX treatment. The Mean ± SD of three biological replicates, with three technical replicates each, are plotted. (B) Recovery time of *M. smegmatis* in 7H9-ADS after different starvation durations in PBS. Mean ± SD of three biological replicates, with three technical replicate each, are plotted. *P < 0.05, **P < 0.01, ***P < 0.001, and ****P < 0.0001; ns, not significant.

We tested whether the duration of the recovery time depends on the duration of exposure and concentration of SHX. We found that the recovery time drastically increased with treatment duration, whereas increasing the SHX concentration had no effect (Fig. 3A and Fig. S3A–D). Interestingly, when subjected to milder stress conditions, such as starvation in a saline buffer, *M. smegmatis* cells displayed consistent recovery patterns, with no variation in the duration of recovery time observed across different starvation durations (Fig. 3B and Fig. S3E). Since the long recovery time following PBS exposure could potentially be due to starvation-induced cell death, we analysed CFU counts after each exposure time. No considerable decline in CFU counts was observed, and the viability remained nearly constant (Fig. S4A).

### Fast-growing *M. smegmatis* cells prior SHX exposure exhibit longer recovery times after treatment

**Figure 4:**
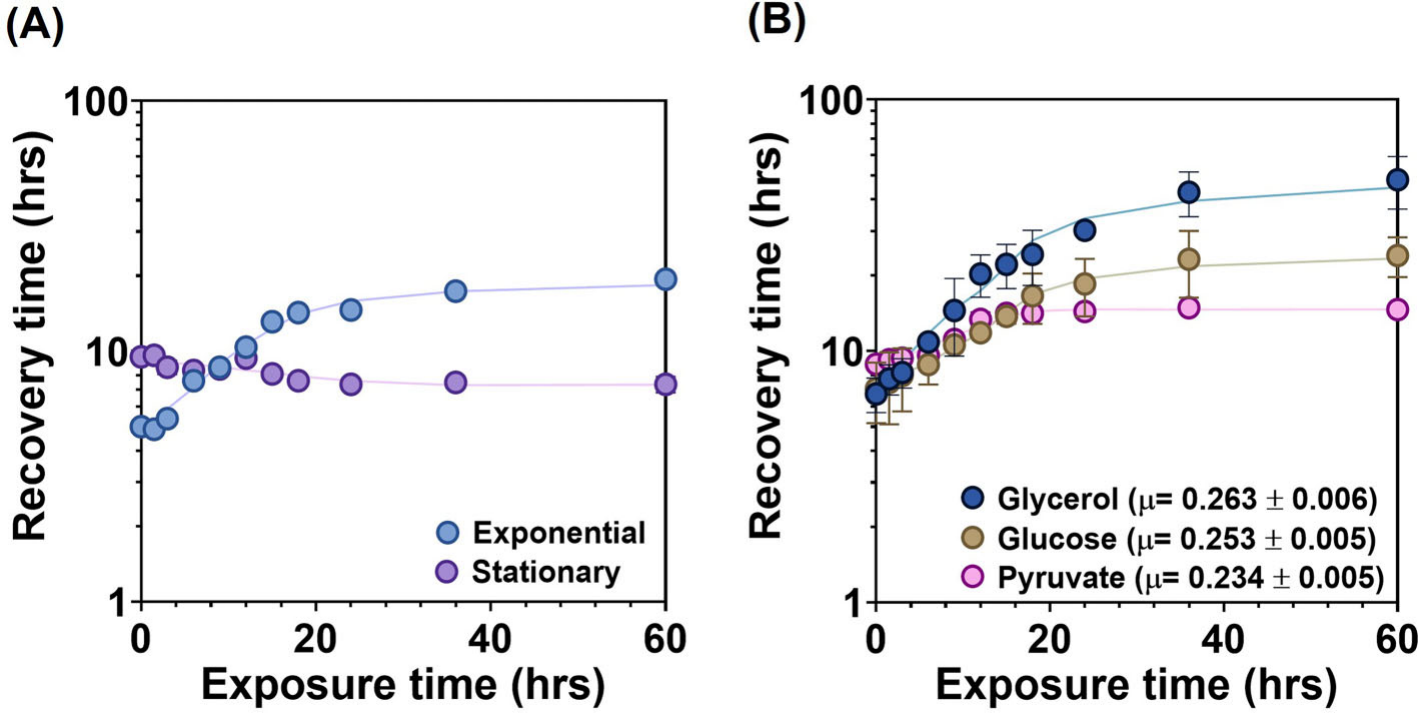
Relationship between pre-growth rate of *M. smegmatis* and recovery time. (A) Recovery time of *M. smegmatis*, growing exponentially or stationary before SHX treatment, in 7H9-ADS after indicated exposure time of SHX. Mean ± SD of three biological replicates are plotted. (B) Recovery time of *M. smegmatis* cells, whose pre-growth rate was varied by growing in the presence of different carbon sources, i.e. glycerol, glucose, and pyruvate before SHX treatment, after indicated exposure times. Solid lines represent smoothing curves added solely as visual guides and were not used for any quantitative fitting or parameter estimation. The Mean ± SD of three biological replicates, each with three technical replicates, are plotted.

We next evaluated how the pre-treatment growth rate of mycobacterial cells influences their response to a SHX exposure, particularly with respect to the recovery time (defined as the time required for population to double its initial optical density after a SHX exposure). For this, we tested the effect of SHX on exponentially growing cultures. We found that the recovery time increased with increased exposure time of SHX (Fig. 4A and Figs S4B and S5A). When compared with stationary cultures, where growth is already halted due to nutrient starvation, the recovery time did not change with exposure time (Fig. 4A and Figs S4C and S5B).

Next, we varied the pre-treatment growth rate by growing *M. smegmatis* on different carbon sources (Fig. 3B). *Mycobacterium smegmatis* grew fastest on glycerol, slower on glucose, and even slower on pyruvate. We found that cells grown on glycerol took a longer time to recover after SHX treatment than on glucose and pyruvate, which support slower growth (Fig. 4B and Fig S4C–E and S5C–E).

### Supplementing regrowth medium with a complete set of 20 amino acids, pyruvate or growth in spent media reduces the recovery time after SHX exposure

**Figure 5:**
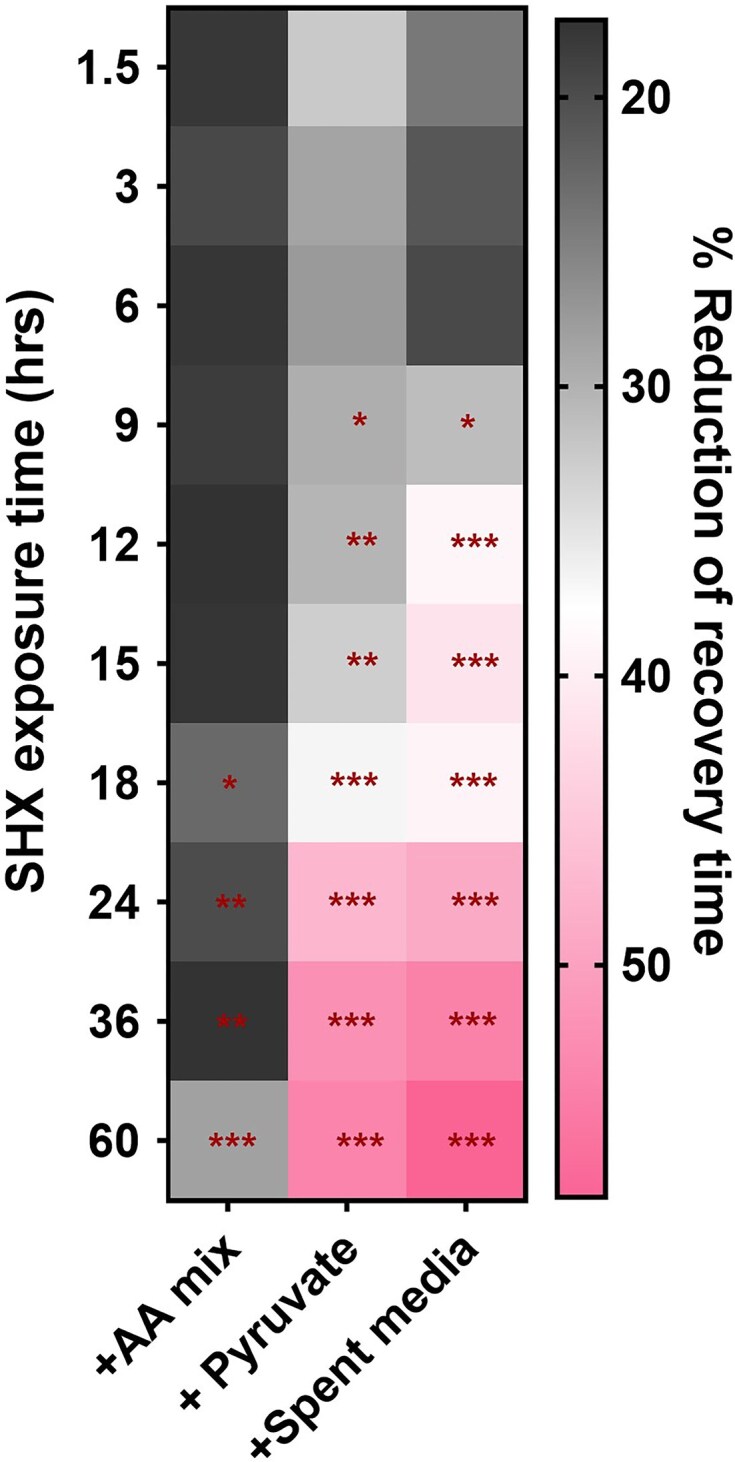
Reduction of recovery time of SHX-treated *M. smegmatis* cells by various stimuli. Heat map representation of percentage reduction of recovery time of SHX-treated *M. smegmatis* at different time points of SHX exposure, with respect to untreated control. SHX-treated *M. smegmatis* cells are recovered for growth either in spent media or regrowth media (7H9 media containing 0.05% Tween-80 added with 0.4% glycerol) supplemented with amino acid mix (+AA, 4 g/L) or sodium pyruvate (10 mM). The scale bar on the right indicates an increase in percentage inhibition. The data are representative of two independent experiments, each comprising three technical replicates. Two-way ANOVA (Analysis of Variance) with Dunnett’s post-hoc test was performed on the raw recovery time (each supplemented condition vs control for each time point). Significance is shown as *P < 0.05, **P < 0.01, ***P < 0.001, and ****P < 0.0001; columns with no asterisk mean not significant.

When cells are exposed to abrupt strong stresses, the cells may end up in a disrupted state that prevents them from mounting an appropriate stress response (Kaplan et al. 2021). Cells may then prove unable to respond, due to, for instance, limiting “bottleneck” metabolites (Basan et al. 2020). We explored the external compounds which, when supplemented to regrowth media, could stimulate faster recovery and reduce the lag time upon removal of SHX. We first tested whether amino acid supplementation of growth media rescues delayed outgrowth of SHX-treated *M. smegmatis* cells. Addition of amino acid mix (AA mix) containing the full set of 20 amino acids to outgrowth media indeed significantly reduced the recovery time (Fig. 5 and Fig. S6A). Whereas for shorter SHX exposure time, addition of AA mix showed only minimal reduction in recovery time (16%–18%), a maximum reduction of 28% was observed for 60 h of SHX exposure (Fig. 5 and Fig. S6A).

Sodium pyruvate has been used successfully as a growth stimulus (Mukamolova et al. 2003, Pinto et al. 2011, Baker et al. 2014), leading to the recovery of bacteria, even from natural environments (Zhang et al. 2021). We tested if its addition to regrowth media could also stimulate faster recovery from SHX stress. After treating with SHX, *M. smegmatis* cells were grown in fresh growth media supplemented with 10 mM sodium pyruvate (Fig. 5 and Fig. S6B). This addition reduced the recovery time significantly with a maximum reduction of 52% observed at 60 h compared with control (Fig. 5 and Fig. S6B).

It has been previously observed that the spent culture supernatant of *M. tuberculosis* improves viability of aged culture (Sun and Zhang 1999). Therefore, we utilized spent media, wherein cells grew exponentially, to regrow the SHX-treated cells. Again, recovery times of SHX-treated cells drastically significantly decreased, with a maximum reduction of 58% at 60 h of SHX exposure compared with control (Fig. 5 and Fig. S6C).

These results indicate that the external chemical stimuli can help the cells exposed to SHX stress to recover faster, possibly by overcoming metabolic bottlenecks.

## Discussion

Survival of mycobacteria under diverse conditions can be attributed to their ability to either form a small subpopulation of persister cells or enter into a population-wide dormant state in response to various stressors (Sarathy et al. 2018). This results in cells characterized by reduced growth and a metabolic downshift, thereby facilitating long-term survival, virulence development, increased antibiotic tolerance, and reactivation of infection (Zhang 2014). Therefore, in recent years, there is a growing interest in studying and targeting of these cells. In this study, using SHX, a stringent response inducing bacteriostatic, we examined how a mycobacterium responds to abrupt growth reduction and recovery. Additionally, we investigated how the pre-treatment growth rate influences the recovery from a sudden environmental stress and whether different external stimuli can fasten the growth recovery.

SHX has proven valuable for studying the stringent response and its role in different stress conditions in various bacteria (Tosa and Lewis 1971, Amato et al. 2013, Pontes and Groisman 2019). However, its effectiveness in mycobacteria has argued to be limited, possibly due to impermeability (Primm et al. 2000), differences in amino-acid metabolism, or insensitivity of mycobacterial tRNA-Ser charging enzyme (Dahl et al. 2003). In contrast to previous studies (Primm et al. 2000, Dahl et al. 2003), and in line with van de Weerd et al. (2016), our study shows that SHX treatment causes growth arrest in *M. smegmatis*. Additionally, we found that these growth-arrested cells exhibit characteristics typical of persister cells i.e. low ATP levels, increased tolerance to antibiotic, and lag in recovery. Our findings suggest that SHX can induce persister-like cells in *M. smegmatis*, likely by activating the stringent response. Therefore, having a tool to activate the stringent response in mycobacteria can be of great practical value and can boost our understanding of its role *in vivo* stress pathway in mycobacteria.

Following SHX treatment, cells exhibited an extended lag time before resuming growth, with increased antibiotic persistence during this lag time and after full growth recovery (till exponential phase) from SHX stress. Similar to our findings, *E. coli* has been shown to recover from SHX-triggered growth inhibition, a process characterized as a resilience mechanism regulated by (p)ppGpp metabolism (Patacq et al. 2020). The increased antibiotic persistence during lag time could be explained by non-/slow growth of population, also known as persistence-by-lag, as antibiotics typically require active growth to be effective. The intriguing aspect was the sustained antibiotic persistence in fully recovered cells, leading to three potential explanations. First, the strong and sudden stress induced by SHX might have prevented cells from activating an effective stress response during treatment. However, upon SHX removal, previously synthesized stress protein mRNA could be translated, providing protection against antibiotics. Second, a subpopulation of bacteria may have entered a dormant state during SHX stress, maintaining this state even upon returning to growth conditions, thereby becoming protective against lethal antibiotics. A third possible interpretation is the phenomenon of ‘response memory’, as described in the literature (Zhang et al. 2023), whereby prior exposure to stress influences subsequent behaviour and enable bacteria to adapt to diverse environmental fluctuations (Lambert and Kussell 2014). While further experimentation is needed to distinguish between true physiological memory and delayed restoration of metabolic homeostasis, SHX treatment may induce cellular states that affect subsequent adaptation to additional stresses, including antibiotics. Recent works have demonstrated various mechanisms of bacterial ‘memory’, including stress-induced protein aggregates that act as non-genetic inheritance modules and persist through cell division (Govers et al. 2018, Bollen et al. 2021), as well as (p)ppGpp-mediated transcriptional imprints and metabolic shifts that can transiently enhance survival during subsequent antibiotic exposure. The dependence of recovery time on SHX exposure duration observed here is consistent with the idea that prior stress history influences subsequent growth dynamics, similar to findings reported by Kaplan et al. in *E.coli* (Kaplan et al. 2021).

However, under less acute stresses, such as starvation in saline, this dependency was not observed, and cells displayed a more uniform recovery time. As previously reported (Kaplan et al. 2021), milder stresses are associated with a narrow range of phenotypes and a more consistent lag time, with exit from growth arrest primarily governed by pathways related to growth (Kaplan et al. 2021). These observations suggest that the phenomenon may depend on the severity and nature of the stress rather than just representing a universal memory mechanism. Nevertheless, further experimentation is needed to confirm these possible explanations.

The ability to vary growth rate allows mycobacteria to adapt itself to different conditions, particularly during chronic infections (Berney and Cook 2010). Therefore, understanding how growth rates relate to recovery times is crucial for strategizing effective drug treatments. We observed that the initial growth rate is important in mycobacterial response to SHX stress. Faster-growing mycobacterial cells exhibited longer recovery times, particularly after long SHX treatment. This is likely because rapidly growing cells, with enhanced cellular processes, are more vulnerable to SHX effects. In contrast, previously slow-growing cells recover more quickly, possibly due to reduced metabolic activity making them less susceptible to antibiotics. The observed correlation between lag time after SHX and pre-growth rate made us wonder about potential metabolic limitations during recovery. While this correlation suggests that pre-treatment growth rate influences recovery, we cannot exclude that carbon-source–specific metabolic differences may also contribute to the observed effects. Therefore, this relationship should be interpreted as a correlation rather than causation, and further work is required to disentangle these effects.

The metabolic limitation, as shown, can be partly alleviated by supplementing growth stimulators. Among the three tested conditions, pyruvate and spent medium strongly accelerated recovery, whereas the amino acid mixture had only a modest effect. This indicates that recovery is not driven solely by amino acid replenishment. In *E.coli*, SHX triggers a transient metabolic overflow, leading to secretion of pyruvate-derived amino acids—alanine, valine, and leucine (Patacq et al. 2020), leaving recovering cells temporarily limited for these intermediates once SHX is removed. Supplementing pyruvate may therefore bypass this limitation, explaining its strong effect. Spent medium from exponentially growing *M. smegmatis* likely contains a broader set of readily utilizable metabolites—including pyruvate and TCA intermediates—that can be immediately incorporated into central metabolism, resulting in similar recovery enhancement. In contrast, the amino acid mixture requires energy-dependent uptake and assimilation, processes that remain constrained immediately after SHX removal. The specific metabolites in spent medium responsible for this effect remain unknown, and further studies are needed to determine whether a similar metabolic imbalance occurs during SHX recovery in mycobacteria.

In conclusion, the interplay between mycobacterial growth rates and recovery times not only sheds light on their adaptability during chronic infections but also underscores the need for a tailored and multifaceted approach to antibiotic treatment.

## Supplementary Material

fnag048_Supplemental_File
