# Supplementary material for: Growth-rate dependent response of mycobacteria to relief of inhibition by a bacteriostatic antibiotic
Source: FEMS Microbiol Lett. 2026 Apr 20;373:fnag048. doi: 10.1093/femsle/fnag048 (PMC13155453; doi:10.1093/femsle/fnag048)
Supplement: fnag048_Supplemental_File [file fnag048_supplemental_file.pdf]

Supplementary information

Growth-rate dependent response of mycobacteria to relief of inhibition by a bacteriostatic antibiotic

Priyanka Chauhan[1], Frank J Bruggeman[1*]

[1]Systems Biology Lab, A-Life, AIMMS, VU University,

De Boelelaan 1087, 1081 HV, Amsterdam, The Netherlands

[*] *corresponding author: f.j.bruggeman@vu.nl*

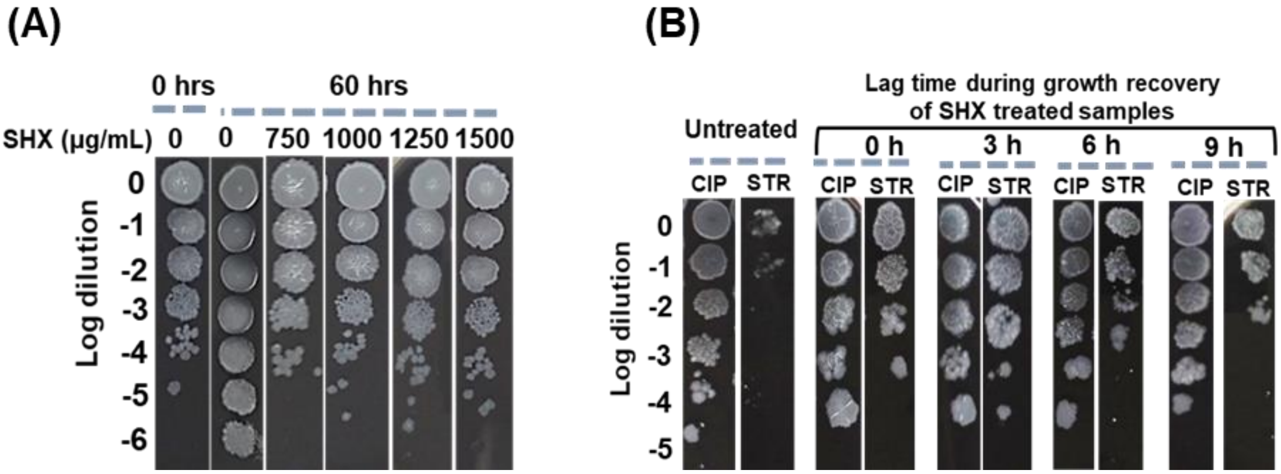

Figure S1: Colony forming unit (CFU) analysis for quantification of surviving M. smegmatis after exposure to **(A)** indicated SHX concentrations for 60 hrs., and **(B)** Ciprofloxacin (CIP) and Streptomycin (STR) at indicated time points of lag time after removal of SHX. Notably, the exposure time (4 hours) for antibiotics was kept shorter than the total lag phase time. The exponentially growing culture without SHX was used as untreated control. Representative image of 3 biological replicates are shown.

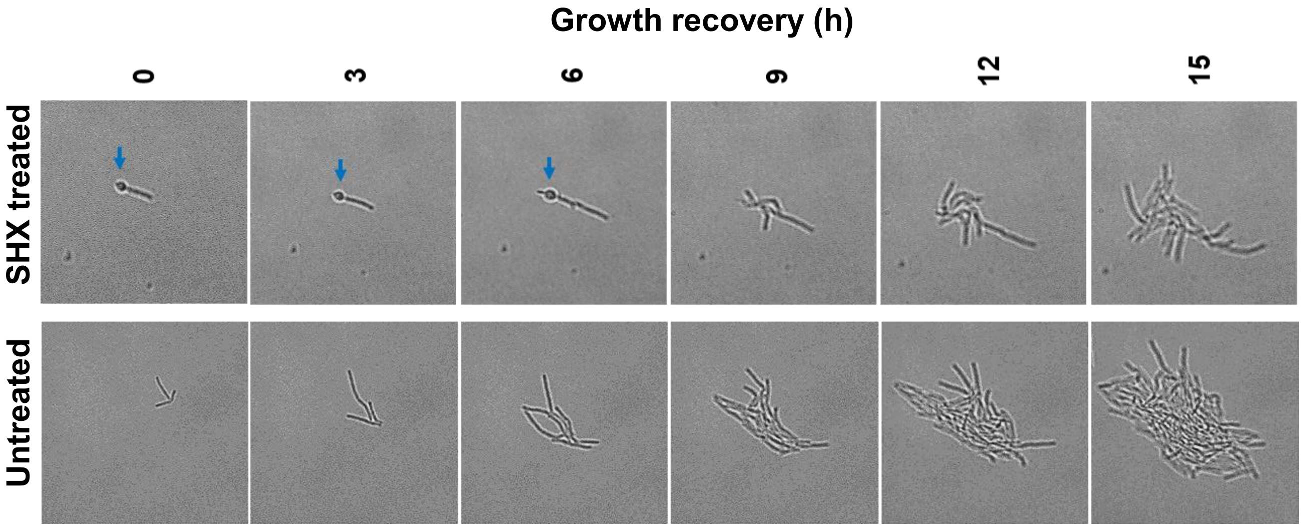

Figure S2: Time-lapse microscopy of SHX treated and untreated culture during recovery phase. The blue arrow indicates the morphological change after treatment with SHX. The figure is representative of 30 untreated, and 37 SHX treated single cell observed till 15 hours in 30 min interval using agarose-pads.

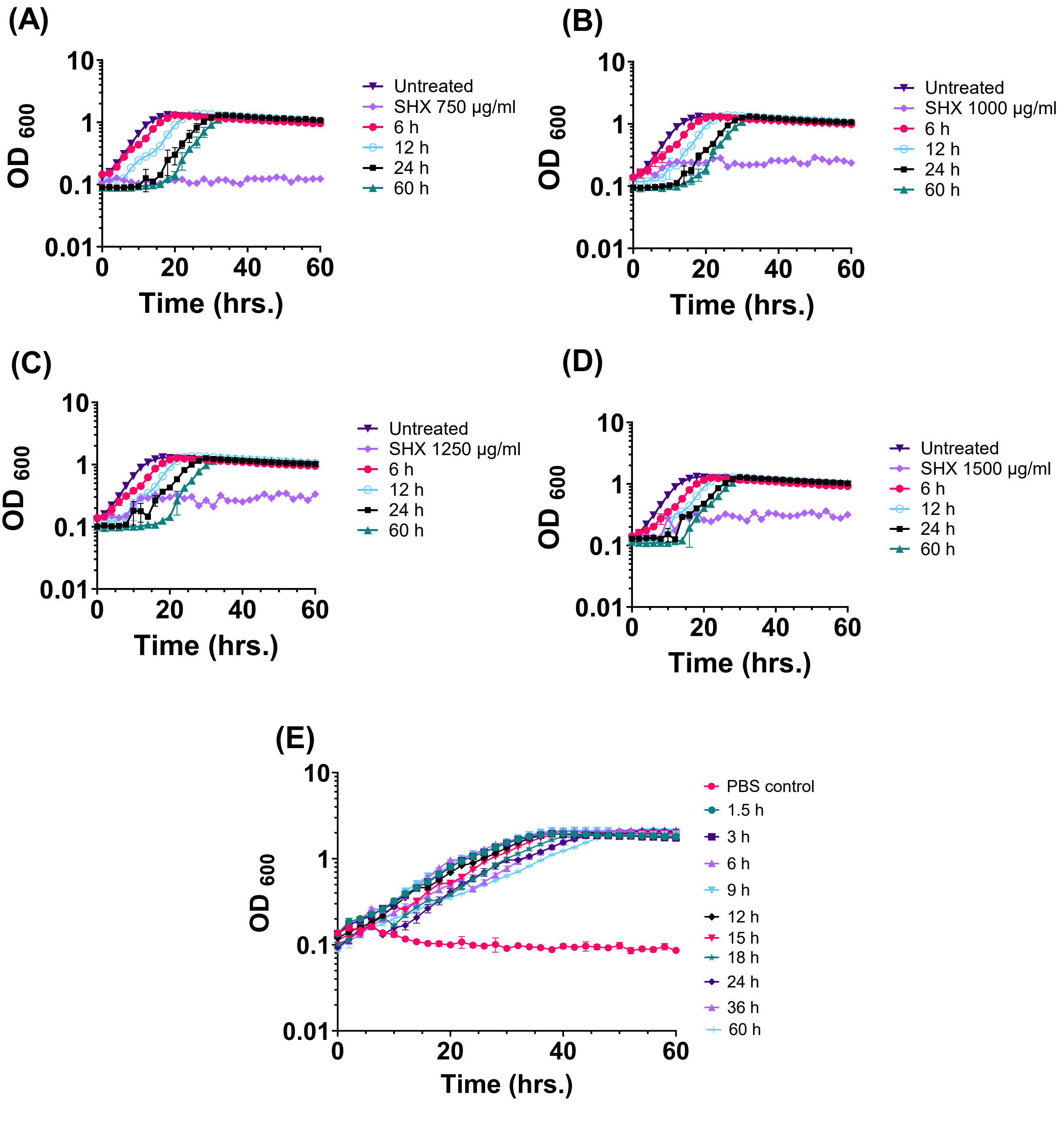

Figure S3: Recovering growth curves of *M. smegmatis* in 7H9-ADS following treatment with different concentrations of SHX: **(A)** 750 µg/mL, **(B)** 1000 µg/mL, **(C)** 1250 µg/mL, and **(D)** 1500 µg/mL, for the indicated exposure times. The “SHX-only” curves for each concentration serve as controls in which cells were continuously exposed to SHX for 60 hours. **(E)** The growth curve of *M. smegmatis* in 7H9-ADS for recovery after different starvation duration in PBS. The “PBS-only” curves serve as controls in which cells were continuously kept in PBS for 60 hours. Three biological replicates were performed, result from one representative biological replicate is shown, Mean ± SD of three technical replicates are plotted.

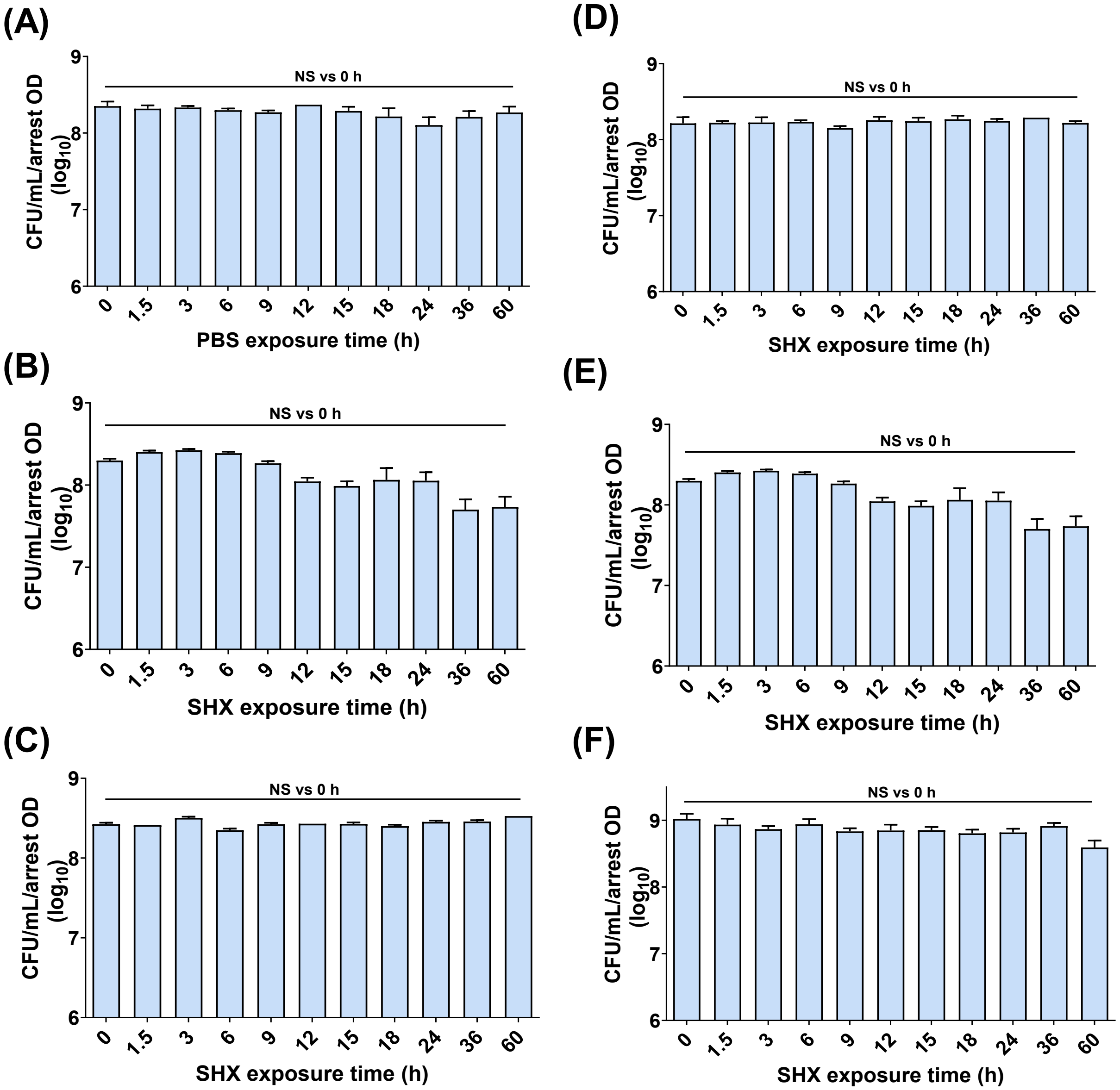

Figure S4: Viability after indicated time of **(A)** PBS exposure, and SHX-treatment of **(B)** Exponential phase, **(C)** Stationary phase, **(D)** Glycerol, **(E)** Glucose, and **(F)** Pyruvate grown culture. The bars and error bars represent the mean ± SD of 3 biological replicates. Nonparametric Kruskal–Wallis test followed by Dunn's multiple-comparison test, comparing each time point to the 0 h control. NS; non-significant

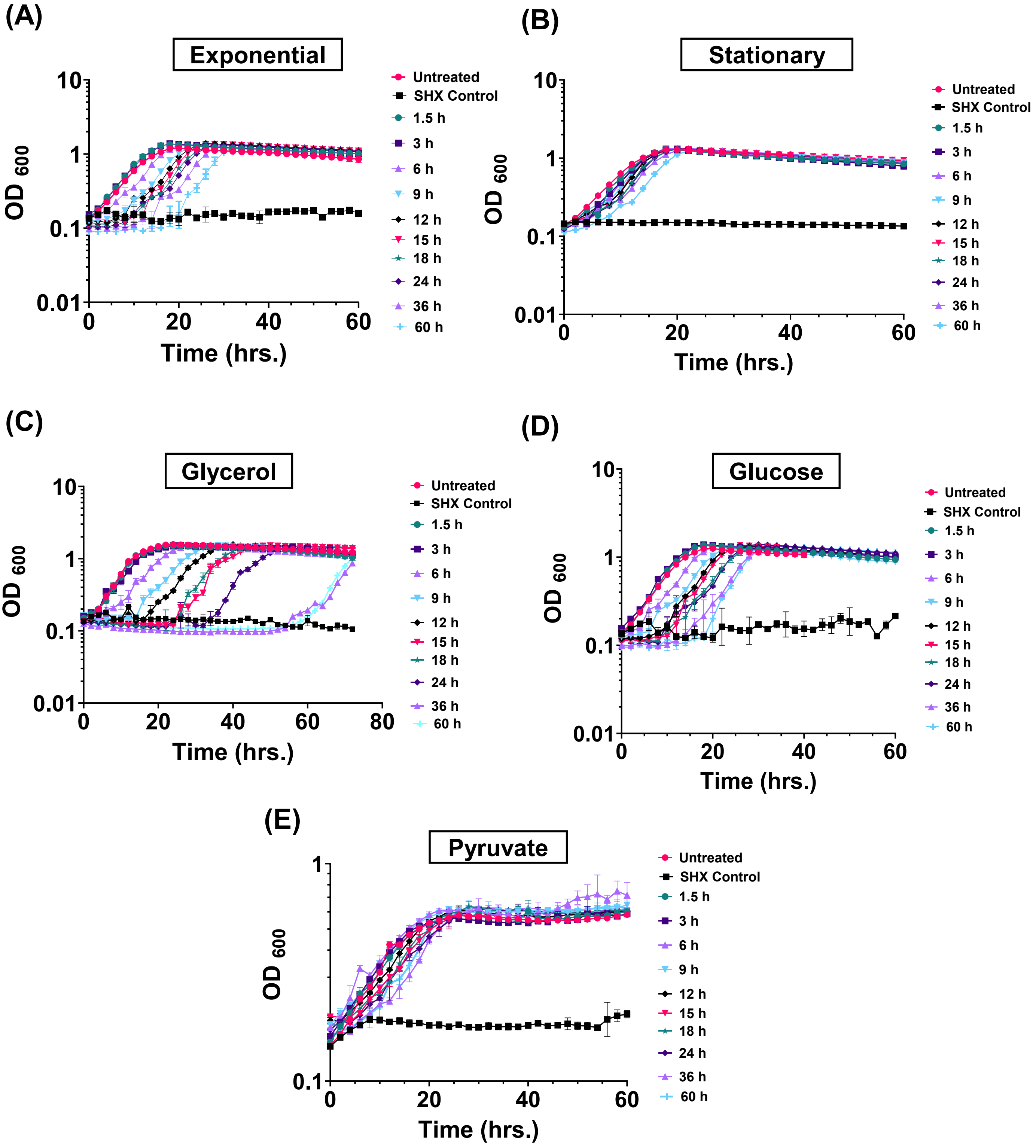

Figure S5: The recovering growth curves of *M. smegmatis,* growing **(A)** exponentially and, **(B)** stationary before SHX treatment, in 7H9-ADS after indicated exposure time of SHX. The recovering growth of *M. smegmatis* cells, whose pre-growth rate was varied by growing in the presence of different carbon sources i.e., **(C)** glycerol **(D)** glucose and **(D)** pyruvate before SHX treatment, after indicated exposure times. The "SHX-control" curves serve as controls in which cells were continuously exposed to SHX for 60 hours. Three biological replicates were performed, result from one representative biological replicate is shown, mean ± SD of three technical replicates are plotted.

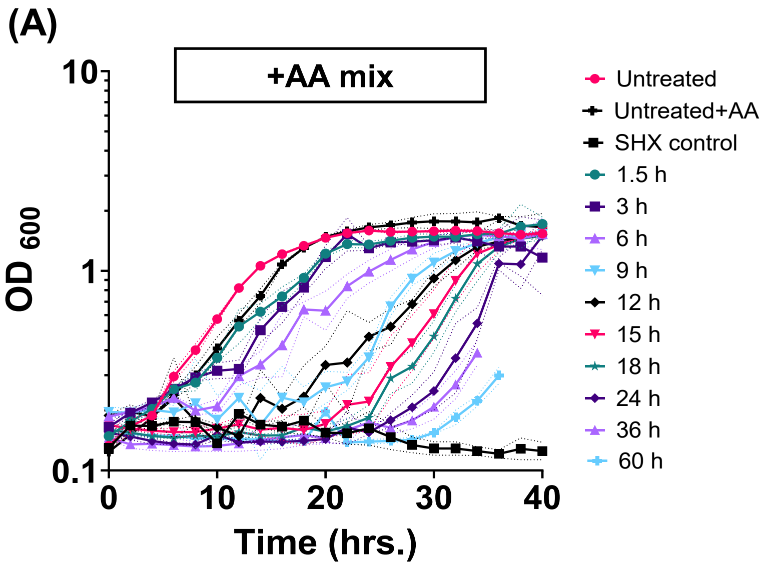

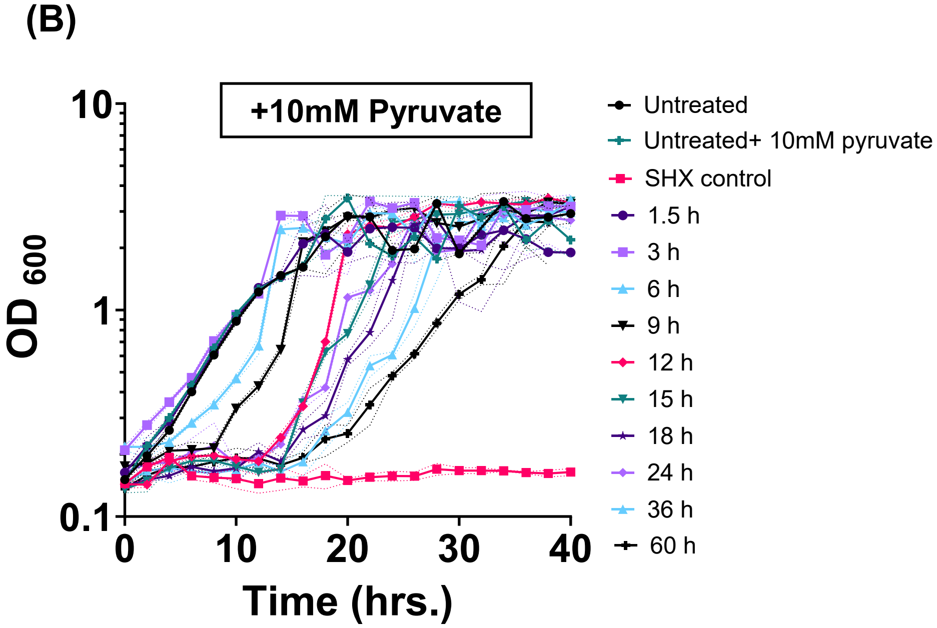

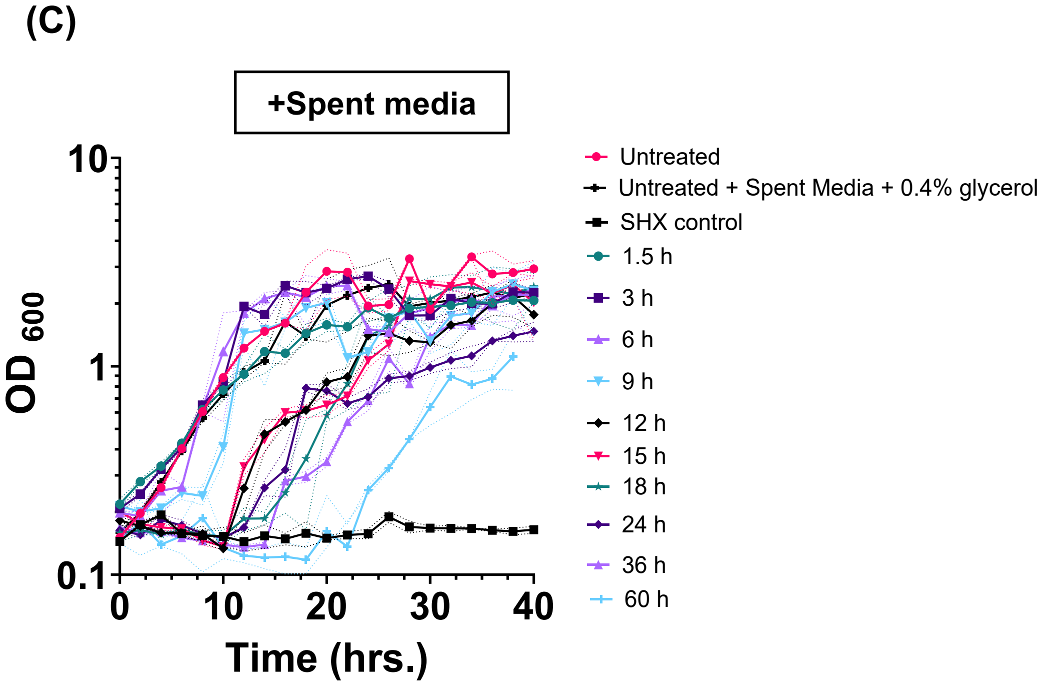

Figure S6: Recovering growth curves of SHX-treated *M. smegmatis* in regrowth medium (7H9 containing 0.05% Tween-80 and 0.4% glycerol) supplemented with either **(A)** amino acid mix (+AA, 4 g/L), **(B)** sodium pyruvate (10 mM), or **(C)** spent medium. The "SHX-control" curves represent cultures that remained continuously exposed to SHX for 60 hours. The "untreated control + supplement" curves correspond to SHX-untreated cultures grown in the same supplemented regrowth media, included to assess whether supplementation alone affects growth rate. Two biological replicates were performed, result from one representative biological replicate is shown. Mean ± SD (dashed line) of three technical replicates are plotted.
